# Adolescents’ healthcare decisional capacity in the clinical context: a theoretical study and model

**DOI:** 10.1016/j.jped.2024.08.004

**Published:** 2024-09-05

**Authors:** Guilherme Henrique Martins, Kalline Eler, Aline Albuquerque, Rui Nunes

**Affiliations:** a Faculty of Medicine, University of Porto, Porto, Portugal; b Federal University of Juiz de Fora, Juiz de Fora, MG, Brazil; c University of Brasília, Brasília, DF, Brazil

**Keywords:** Decision-making, Adolescent, Bioethics, Clinical ethics

## Abstract

**Objective:**

To provide a theoretical study and model for the bioethical foundations of the factors that influence adolescents' healthcare decisional capacity.

**Sources:**

Materials from diverse sources, including indexed articles in recognized databases and official government documents, were examined for a purposefully selected sample. The research consisted of two stages: selection of documents and reflective thematic analysis, followed by the preparation of a report. The analysis adopted a phenomenological stance and a reflective view compatible with human rights. To reduce bias and ensure the robustness of the results, measures such as data triangulation were employed. Ethical measures were taken to ensure data integrity, including considerations of anonymity and conflicts of interest in the selected studies.

**Summary of the findings:**

It was possible to list intrinsic and extrinsic factors of the adolescent patient that influence their decisional capacity regarding health. A theoretical model was developed to discuss these factors for evaluation by means of an infographic.

**Conclusions:**

It seems clear that the evaluation of healthcare decisional capacity of adolescents must position itself ethically regarding the tension between the moral duty to respect the self-determination of the able subject and the need to protect adolescents decidedly unable to make a specific health decision at a given time.

## Introduction

Commonly, the patient's healthcare decisional capacity is evident, and health professionals evaluate this ability when exercising care if necessary, based on the assumption of its fullness.^1^ When in doubt, the health professional should use validated technical instruments to determine how capable the patient is, preferably, but not limited to, in a hospital environment.^2^ This need originates from particular health conditions such as decisions made in notably adverse emergency situations, or by patients with neurodevelopmental disorders, congenital neurological conditions, or in situations of exacerbations of psychiatric conditions such as bipolar disorder or schizophrenia.

In a clinical healthcare context, much is discussed about the need to promote patient autonomy and its empowerment surrounding decision-making. From a bioethical perspective that recognizes the subject's self-determination as a human right that should not be replaced by paternalism, the understanding of how capable the patient is to make their own decisions, that is, their healthcare decisional capacity, has been gaining relevance.^1^^,^^3^ Deliberation in the context of healthcare includes the decision to start or stop clinical or surgical treatments, the performance of examinations, or even hospitalizations. Situations in which adolescents wish to maintain confidentiality regarding their care are not excluded from this perspective, even if the participation of parents or guardians is encouraged by the health professional, such as in cases of drug use, prescription of contraceptives, prenatal supervision and/or in the diagnosis of infectious diseases.

The expression *decisional capacity*, also called mental ability or competence, refers to the skills that are necessary to make any decision. In turn, *healthcare decisional capacity,* or health capacity, is the ability to make decisions about healthcare.^3^ In an Anglo-Saxon legal context, the concept derived from the literal translation of *capacity* differs from that of *competence*, because the latter appears in the legislation of those countries as a form of skill for various life issues. Therefore, it could not be deliberately expanded as a synonym for the former when addressing the issue of decision-making regarding health.^4^ In addition, still in the conceptual field, a given individual can be considered capable of making decisions but, at a given moment, for a passing reason, incompetent.^5^ The bioethical understanding adopted by the present theoretical framework uses only the term capacity and, therefore, this word is chosen in this document in an attempt to simplify the terminological problem.^1^^,^^3^ That said, considering the premise of guaranteeing the autonomy of the individual, the evaluation, that is the qualification, of the healthcare decisional capacity of a patient gains notoriety and becomes highly necessary from a bioethical point of view since, only by weighing its definition and exhausting the support for decision-making, one can restrict the right to self-determination.^1^

Although vulnerable due to their developing maturity, children and adolescents also hold human rights, a point that has been established among scholars in the area.^6^ For this reason, the principles under discussion are those contained in the theoretical-normative framework of the Convention on the Rights of the Child (CRC)^7^ which, with binding force on the signatory States of the United Nations (UN), has been impactful, especially on healthcare practice. This document brought to the debate the fact that the child is not an object of protection and property of its parents/guardians, but rather a subject with rights.^8^ It is worth noting that, according to this same CRC, all human beings under the age of eighteen are understood to be children and, although the CRC does not directly explain the concept of adolescence or adolescence, subsequent documents do so indirectly – namely, General Comment No. 4 and General Comment No. 20 of the document, which deal with the health of the adolescent and their rights.^9^^,^^10^ Such publications categorize this group between the ages of ten and eighteen. It is then clarified that, due to the interest of this document in expanding discussions at the international level, given the biological, psychological, and social particularities of the public in question, it focuses on children between twelve and eighteen years of age.

By adopting the theoretical-normative framework of the Convention on the Rights of the Child – CRC,^7^ the adolescent is then considered as a being whose capacities are *evolutionary capacities* - according to articles five and twelve of the CRC itself. The fact that the authors consider these individuals in a constant process of biological, psychological, and social maturation directly impacts the understanding that, if adolescents is a being under construction, their abilities are constantly changing due to a gradual maturation of the central nervous system and, therefore, of their various abilities.^5^ The main consequences of this understanding fit the fact that children are considered to have temporarily increased vulnerability when compared to adults, as well as that their decisions should be given due support to guarantee their right to participation, information and privacy in healthcare.^8^

Health professionals evaluate a patient's decisional capacity to answer the following question: *should the patient's will and preferences be respected?* This work proposes a new focus and suggests: *is the patient sufficiently capable of making a specific decision at this time?* This question is very much directed towards casuistry, especially regarding the nature of the decision, the subject, in line with the idea that the healthcare decisional capacity of adolescents would be fluid, mobile, and singular, in addition to being an aptitude sensitive not only to biological factors but also to environmental and social factors.^8^^,^^11^ Enjoying the fullness of one's capacity, even if the light of biomedical knowledge seems irrational, the decision is nonetheless legitimate from the human rights standpoint. The same could not be said if one's healthcare decisional capacity were debatable, and therein lies the gap that this work proposes to help fill, from the perspective of the factors that influence it. Expert literature compares the assessment of decisional capacity to the magnitude of a court decision, given that in order for it to be delivered, an assessment of decisional capacity often needs to be made to support it.^2^ The same author states that despite the general recognition of the need for criteria to assess the decisional capacity of a patient, including, but not limited to adolescents, there is a divergence in the literature on which criteria should be included in this process and, therefore, a gap to be filled by academic research.^2^ In the case of a group of patients whose personal perspectives and understandings are routinely disregarded due to their presumption of incapacity - in the case of children/adolescents, there is an increase in the difficulty of assessing aptitude for the decision.^8^

*Healthcare decisional capacity* for decision in a clinical context is known to be a complex issue and this article does not intend to exhaust this discussion, precisely because the authors recognize the importance of the topic. That's why it is necessary to clarify that it is not the authors' intention to address applications in specific contexts such as in cases of terminality, palliative care, euthanasia, use of contraceptives, abortion, teenage pregnancy, or vaccination. In order to fill a gap in the predisposed dichotomy between capacity and disability, it is necessary to discuss the assessment of healthcare decisional capacity in order to ensure the realization of the right to self-determination of the adolescent patient. Therefore, the objective of this study was to provide a theoretical study and model for the bioethical foundations of the factors that influence *adolescents’ healthcare decisional capacity*.

## Methods

This is a theoretical, documentary study, based on the principles of the CRC and the references of Albuquerque^3^ and Eler,^8^ and originated from the interest in clinical bioethics shared among its authors, based on their activities in the respective educational and research institutions to which they are linked.

The content of books, articles from academic journals, and institutional documents of interest were analyzed, such as those linked to databases qualified by secure bibliographic metrics, including Virtual Health Library Brazil (VHL Regional®), Online System for Search and Analysis of Medical Literature (MEDLINE®), Science Direct®, Scopus®, Web Of Science®, and websites of international institutions such as the World Health Organization (WHO) and its related agencies. The total number of documents studied was composed by theoretical intentional sampling.^12^ Saturation by document search was achieved when consistent insight into the assessment of adolescent healthcare decisional capacity was developed, and when researchers stopped gaining new knowledge after collecting new data.^13^

The search was performed in two steps, followed by the preparation of a report, namely: document selection and reflective thematic analysis.^14^ The documents were selected according to the scientific qualification of four factors: authenticity, credibility, representativeness, and meaning.^15^ Reflective thematic analysis was done in order to consider the subjectivity of the investigator as a scientific resource when discussing the bioethics, phenomena, events, and concepts found from the perspective of human rights.^14^ The agreement on this perspective made it possible to resolve any differences between the authors. The authors chose to use an epistemological stance of the phenomenological type when analyzing the documents and their contents, inferring objective and subjective interpretations of them.^15^ A reflective view was adopted, compatible with the idea that bioethical problems relate to various sociocultural contexts and, therefore, it is also up to human rights to discuss the confrontation of such situations.^16^ Results are presented regarding the *factors that influence* and the *assessment of adolescents’ healthcare decisional capacity*.

In order to reduce the impact of possible biases in the studies on which this research was based, information collected through several methods, including qualitative and quantitative approaches, was thoroughly analyzed. Thus, a triangulation of the data was promoted, generating empirical inductive knowledge, and ensuring that the data discussed did not come from a single scientific perspective.^12^ It should be noted that the use of pre-existing documents as a form of raw data to be analyzed raises relatively fewer ethical concerns than other research methods, since the records are generally in the public domain and, in the case of a sample composed of human beings, are protected by anonymity. In the case of authors of books and articles published in journals and specialized magazines, it is known to them that their production will be available to the academic community and, thus, subject to constant challenge at the methodological level and in terms of the content itself. In order to mitigate possible conflicts of interest in the documents studied, the researchers listed only studies whose funding was well delimited to the parameters of scientific integrity.^17^

### Factors influencing adolescents’ healthcare decisional capacity

It is not an unprecedented fact that data regarding the assessment of healthcare decisional capacity of adolescents is limited and that this study aims to enrich this discussion. In this sense, from a reflective stance, it seemed natural and feasible to gather knowledge from sources beyond those related exclusively to clinical bioethics, establishing reasonable connections with other areas of knowledge. For this reason, many studies listed below do not belong to the restricted scope of clinical bioethics and the appropriate proportions must therefore be kept for their application in healthcare. Other studies, however, present a greater connection to healthcare and present themselves more explicitly as pillars of the determinants of the adolescent healthcare decisional capacity audience. Additionally, the decision to extract factors influencing healthcare decisional capacity from the profile of decision-making during adolescence is clarified, reflecting decisions in the manner detailed below.

Starting the discussion about the factors that influence *adolescents’ healthcare decisional capacity*, it is necessary to demystify some findings rooted in common sense. A broad literature review presents empirical findings that discuss facts hitherto considered speculations on the subject:^18^

(a) The risky decision-making behavior during adolescence is associated with the morphofunctional changes characteristic of puberty – and this does vary with age. In other words, decision-making in adolescents is associated with a physiological phenomenon rather than a pure and simple chronological age. This consideration alone brings to the discussion the need to understand adolescents' decision-making as possible as the exercise of a right, considering their will and preferences.

(b) Adolescents become progressively more capable and less vulnerable from the age of fourteen/fifteen - a situation in which puberty, physiologically, is a past stage of development. This demonstrates that there is a biological component in the adolescent's healthcare decisional capacity. However, it is not unique or isolated but part of a set of factors that the authors wish to present here.

(c) Any decision-making during adolescence involves not only neurobiological processes but also interactions with environmental factors, including familial, social, economic, and cultural aspects.

(d) There are two thought-to-be explanatory neuroanatomical and physiological models of decision-making during adolescence: the *socioemotional*, whose location is limbic and paralimbic, and the *cognitive control*, whose location is prefrontal cortical.

Regarding specifically the age at which the individual would be able to make clinical decisions. Adolescents could be prepared to make clinical decisions from age twelve, but puberty as a biological phenomenon could cause some instability.^5^ Despite representing a consistent summary of information on factors associated with deliberation in matters of health, the authors were unable to detail their determining factors, maintaining a gap on this subject. According to the Swiss Academy of Medical Sciences, unlike this factor, it seems clear that emotional and motivational factors, as well as those related to the formation and realization of individuals’ will, should be considered during the assessment of decisional capacity.^19^

No studies were found whose primary objective was to compare healthcare decision-making between male and female adolescents. However, some did so as secondary objectives and some considerations in this regard can be outlined. It is possible to affirm that the influence of gender does not seem to lie only in the fact that one makes more acceptable decisions than the other but is correlated with other factors. Using a psychological educational game with a psychometric purpose, it was found that boys are more likely to make risky decisions when they are with peers of the same age and sex.^20^ There seems to be some level of correlation between the encouragement arising from the presence of a colleague in decision-making and, therefore, it can be concluded that the environment in which the adolescent is situated, regarding the company, directly influences how they make decisions, and it is therefore postulated as a determinant of their decisional capacity on a specific subject at a specific time. In order to assess how moral decisions relate to some adolescent characteristics, a recent study found, for example, that male adolescents were more likely to perceive such challenges as morally acceptable compared to female adolescents.^21^ It is not yet possible to identify the magnitude of the gender component in the assessment of decisional capacity, but it does not seem premature to state that it is one of its determinants and should be taken into account in its assessment.

In Surat, India, through an instrument called *General Decision Making Style*, a study analyzed characteristics of decision-making among adolescents aged seventeen and nineteen (*n* = 1117).^22^ The scale with twenty-five assertions about individuals' perceptions of their decision-making was considered statistically reliable and generated five patterns of decision-making: individual intuitive, rational, dependent, avoidant, and spontaneous, according to the averages of the responses. It was noted that decision-making was better if participants lived with their parents, had a more educated legal guardian, and if they were pursuing a college career themselves. In the same study, those who had a better perception of their self-esteem, creative thinking, and better aptitude for problem-solving were considered more apt to make decisions, which speaks in favor of the importance of factors intrinsic in adolescents that influence decision-making and, therefore, the evaluation of their healthcare decisional capacity. In this sense, the factors associated with physical, emotional, and moral development, which comprise what is called maturity, would be directly proportional to sound decision-making.^23^ Personal experiences were also included, another determining factor intrinsic to the adolescent, associated with emotion in decision-making.^24^, ^25^, ^26^, ^27^ Health literacy is defined as the degree to which individuals can find, understand, and use information and services to inform health-related decisions and actions for themselves and others.^28^ This appears to be another factor that has a direct impact on the ability to make decisions: specialized literature suggests the possibility of this ability improving decision-making processes, making them more effective, as is the case with decisions relating to the reproductive health of adolescents with cancer.^29^^,^^30^

Their history of general and specific health conditions seems to be another determining factor for decision-making regarding health and, therefore, should be considered in the assessment of the healthcare decisional capacity of adolescents. In a robust field research comparing psychological factors of adolescents with temporomandibular disorder, with or without a history of facial trauma, it was concluded that the presence of facial trauma causing the disease negatively influences decision-making in that group.^31^ The authors listed psychological factors associated with the history of trauma, such as phobic ideation, somatization, and hostility, most present in patients with a history of facial trauma to justify their difficulty in decision-making.^31^ From the perspective of decision-making and potential losses and gains during the teenager's life experience, it is seen that the greater the severity of the situation, for example, mistreatment, the greater the difficulty in decision-making.^32^

Considered by this work to promote the individual characteristics of the adolescent patient, culture appears to present more robust data regarding its influence on decisional capacity among adolescents. In a field study with adolescents living with HIV/AIDS, it was found that religiosity moderates decision-making in adolescents, precisely to the detriment of the finding that they were individuals up to four times more likely to maintain adherence to morbidity treatment.^33^

Some theoretical advances are well established when the role of professional support in the assessment is the subject, as well as the presence of subjectivity of the rater during the assessment process. When pronouncing a status of disability, the evaluator is influenced by their own values and standards, as well as by the values and standards of the society in which they are embedded, and these factors must be mitigated to maintain the maximum smoothness of the evaluation process.^34^ In this scenario, it is unacceptable to conclude that a patient is incapable when a decision is not in accordance with the recommendations or opinion of the evaluator, and there are no records of guidelines that establish ethical standards that support isolated expert opinions.^19^

The *adolescent healthcare decisional capacity* is, therefore, the result of the sum of determinants that are directly or indirectly connected to adolescents themselves, forming a complex system that influences the decisional status of the patient, at a given time, under a given decision. Thus, these factors are classified into factors intrinsic to the patient and factors extrinsic to the adolescent patient, both acting synergistically in a dynamic model that should be considered in the evaluation of healthcare decisional capacity in a clinical context, and that is summarized in Table 1.

**Table 1:** Factors influencing adolescent healthcare decisional capacity.

**Intrinsic factors of the adolescent patient**
Age, gender, cognitive and pubertal development, maturity, health literacy, life experiences, history of general and specific health conditions.

**Extrinsic factors of the adolescent patient**
Family support, support from healthcare professionals, environmental conditions (culture, housing, social relationships).

### Assessment of adolescents’ healthcare decisional capacity

Much of the discussion in the expert literature regarding the assessment of the adolescents’ healthcare decisional capacity is rooted in principles similar to those applied to adults: respect for the autonomy of those who can make decisions and for the protection of the human rights of those with temporary or permanent disability.^2^ Laying the theoretical foundations of this subject, Appelbaum and Grisso^35^ identified four key components of decisional capacity, forming what they termed the 'four-skill model': understanding, appreciation, reasoning, and expression of choice. *Comprehension* involves the ability to understand information, necessitating effective communication with healthcare providers; *Appreciation* refers to the ability to manipulate information, promoting cost-benefit relationships, and action and reaction/consequence; *Reasoning* involves the ability to apply logic to the situation and make informed assessments; Finally, communication refers to the ability to articulate decisions among available options. The analysis of this skill should consider that verbal communication may not always be possible in certain clinical settings.^2^ This model is not exempt from criticism and can be considered, although widely used, hyposufficient by disregarding issues such as the patient's values and the impacts of their decision on social life, to the detriment of a more “cognitive” decision.^36^ Consequently, there is an attempt to seek a more modern, context-sensitive, multifaceted approach to assessing healthcare decisional capacity, moving away from the previously advocated 'hypercognitivist' approach.^34^^,^^37^

Based on the emerging perspectives given by Appelbaum and Grisso,^35^ subsequent studies were gradually incorporated into the theme, aligning closely with their initial findings.^38^, ^39^, ^40^, ^41^, ^42^, ^43^, ^44^, ^45^ Addressing issues specific to adolescence, the specialized literature delineates six key aspects constituting healthcare decisional capacity: understanding the object of the issue; analyzing the consequences of their choice, weighing the risks and benefits; judging the information in light of their own values; claiming a certain result with their actions; communicating their desires, fears and doubts in relation to the object of an issue and communicating their decision in relation to the object of an issue in an understandable and coherent way.^46^ Authors also stress the importance of understanding the intentions behind decisions, a critical factor to be considered during the evaluation of decisional capacity as it reflects both the active attitude of the adolescent patient and their pre-decisional deliberation concerning health-related choices.

Theoretical studies have expanded the assessment of decisional capacity with three preliminary steps preceding the four-skill model: (1) addressing communication limitations that hinder the patient's ability to express themselves, encompassing not just verbal issues but also hearing and visual impairments, and dysarthria; (2) evaluating and addressing reversible acute organic causes of disability such as infections, adverse effects of legal and illicit substances, cerebral hypoxia, metabolic disorders and multifactorial delirium; (3) considering the patient's cultural dimension.^4^ Similarly, in preparation for assessing healthcare decisional capacity in adolescence, a structured approach in five steps has been proposed: (1) re-evaluating the patient's clinical and personal circumstances to reaffirm therapeutic indications; (2) optimizing the environment and support conditions to facilitate adolescent decision-making; (3) confirming the patient's understanding of their clinical situation; (4) organizing decisional factors based on thresholds and gradients of decision and, finally, (5) assessing the patient's capacity.^47^ It should be emphasized that, by thresholds and gradients, the authors refer to questions, such as age, diagnosis, risks and benefits described in the literature (limits), patient maturity, previous experiences, psychological conditions, and personal goals (gradients).^47^ Regarding these collaborations in the assessment of decisional capacity, it's important to note that these frameworks emphasize a holistic view of health-related decision-making, demonstrating that cognitive functions alone do not determine capacity; extrinsic factors also play a crucial role, complementing rather than replacing those identified by the *four-skill model*.^44^

In this sense, why is it difficult to make progress in assessing children's and adolescents’ decisional capacity? This important guiding question in discussions surrounding healthcare decisional capacity, which also encompasses adolescents, stems from several studies conducted by Professor Irma Hein and collaborators.^48^^,^^49^ The issues raised by the authors include a) normative aspects, where many countries' legal systems associate healthcare decisional capacity with age; b) biological aspects of neurodevelopment, as adolescents may lack the extensive knowledge of adults, which limits their ability to apply cognitive capacity to concrete problems; and c) the lack of standardized instruments to characterize healthcare decisional capacity. It is crucial to note that all proposed models for understanding decisional capacity emphasize the importance of both verbal communication and the comprehension of written information. However, these models often target adults and may not effectively accommodate the perspectives of adolescents, posing a barrier that needs to be overcome with necessary adaptations.^50^

**Figure 1:**
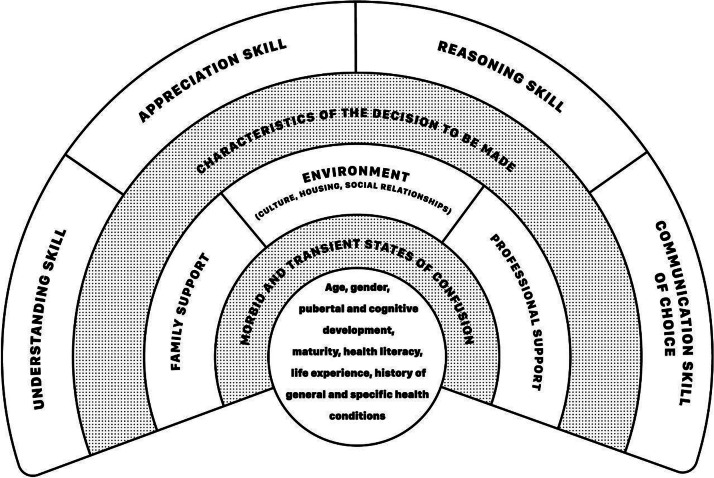
Technical evaluation model of *adolescent healthcare decisional capacity* in the context of healthcare.

To explain the factors that should be considered in the technical evaluation of *adolescent healthcare decisional capacity*, this study proposes a theoretical model expressed through an infographic, organized in layers and inspired by the *Dahlgren and Whitehead Model* of social determinants of health, with which it shares only the spatial configuration of the design.^51^ In the model proposed here, the adolescent patient is centered around the care, and from the center to the periphery, the determinants that can influence the skills involved in the process of evaluating healthcare decisional capacity specific to this group are described and must be taken into account during the evaluation (Figure 1). The model does not translate into a step-by-step guide and is not intended to do so, but rather to serve as a conceptual framework for assessing adolescent healthcare decisional capacity, providing a foundation for its qualitative assessment or for the development of instruments that can quantify it. By considering the particularities of each factor and mitigating any impediments, the evaluation reaches the *four-skill model*.^44^

In the center of the infographic are intrinsic factors of adolescents' health decisional capacity, namely: age, gender, cognitive and pubertal development, maturity, health literacy, life experiences, and history of general and specific health conditions – including any chronic morbidities. Although life experiences stem from interactions with the environment, during the evaluation of healthcare decisional capacity, these experiences represent a summary of what the adolescent has lived so far and, therefore, constitute an intrinsic component of their identity. The specific issue of age has been previously discussed and, for the purposes of this study, it is considered that adolescents between fourteen and fifteen years of age can make decisions equivalent to those of an adult.^18^

Then, it is up to the evaluator to consider an additional factor: a test to determine if there are any situations that may transiently cause confusion in the adolescent patient, thereby limiting their healthcare decisional capacity, even if only temporarily. The necessity of this factor is justified by the fact that, for example, an infection with systemic repercussions might impair the adolescent's understanding when they are required to make an important decision about their own health. In such cases, until the situation is resolved, the adolescent's decision-making capacity would justifiably be deferred. In this scenario, is the responsibility of the health professional, as far as possible, to mitigate these limitations after identifying them, thereby promoting the adolescent's autonomy. Similar scenarios could arise temporarily or permanently, due to adverse effects of licit and illicit substances, cerebral hypoxia, metabolic disorders and multifactorial delirium.^2^^,^^4^ Decision-making in these situations is directly associated with the potential physical or psychological harm that may result from the decision. Health decisions with less impact, which are discussed further below, can still be made based on respect for the patient's self-determination, even if they appear "irrational" from the perspective of an adult or health professional. For example, a patient affected by delirium, who can only murmur incoherently, demonstrates an inability to meet the criteria established by the *four-skill model*, the final step in the evaluation model for healthcare decisional capacity presented in this work.^52^ Therefore, it is essential to emphasize that adequate decision-making in healthcare involves a comprehensive decision based on the patient's wishes and preferences, ensuring their human rights. Highlighting that a full decision is not just a presumably rational decision for an adult with other perspectives and values.

Beyond the central layer of factors intrinsic to adolescent patients that influence their healthcare decisional capacity and considering any temporary extrinsic factors that could destabilize them, there are additional external factors that may affect their decisional capacity: family support, the support of health professionals and environmental issues in which the individual is integrated. These factors are independent of the adolescent's will or preferences. For example, a vulnerable adolescent might be part of a dysfunctional family dynamic or lack stimulation for adequate neuronal development, making them feel unsafe to make decisions. In this context, issues such as financial concerns also play a role, not only affecting adults in productive lives.^52^ Additionally, it is known that adults, including parents and health professionals, significantly influence the decision-making efforts of patients in this age group, acting as facilitators in the process.^53^

From the point of view of the children's healthcare decisional capacity - not specifically adolescents - a model of four clusters of predisposing factors for health-related decision-making was proposed by the academic environment, particularly in research on their participation in clinical trials.^54^ These clusters are the patient (cognitive development and personal experience); the parents (specificities of their relationship with the patient); the physician (characteristics and quality of their relationship with the patient, engagement with the situation, and empathy with decision-making interlocutors); and the situation (severity of the issue to be decided). The similarity between the model proposed by this work and the cited model by the authors is limited to the factors of patients, parents, and physicians (or other health professionals), even if in different positions and activities. Additionally, the proposed theoretical model includes an important addendum: morbidities, whether acute or chronic, manifest in a very particular way depending on specific conditions that may influence the healthcare decisional capacity of an adolescent. This situation is exemplified by an established diagnosis of acute nasopharyngitis of viral etiology may resolve spontaneously in an individual without risk factors for complications, while for others, a pharmacological intervention is essential. This highlights the role of the evaluator of the adolescent's healthcare decisional capacity, who, with their professional and potentially personal experience, can stimulate decision-making and promote the adolescent's ability to do so whenever possible.

Based on this assumption, while it is not the primary focus of this work, it is important to acknowledge the significance of this area in clinical bioethics research that can use an instrument called *decision aids*.^1^ The maturity factor also comes into discussion, directly proportional to the intrinsic conditions of the adolescent patient, showing the interdependence between the layers proposed by the present theoretical model. This includes the level of psychological functioning of the patient's mental structure.

The relationship between the individual's maturity and the object in question operates on a "sliding scale", presenting a thorny and still debatable point from the perspective of the authors of this article: the assessment of decisional capacity should be more rigorous as the severity of the probable consequences of the decisions increases.^52^ There is also an obscure zone between the extremes in which a capacity status is discussed, such as when a partially disabled individual makes a moderately risky decision. In these cases, as previously mentioned, the capacity of the health professional and the patient's support network is crucial to promote autonomy while respecting the patient's will and preferences.

Finally, considering the factors that influence the adolescent's healthcare decisional capacity, there are the skills of understanding, appreciation, reasoning, and expression of choice, as previously mentioned, considering its strengths and limitations.^35^ This model encompasses a set of factors that precede, rather than replace, the *four-skill model*. Even though it is designed to be as broad as possible, the potential limitations to its application cannot be ignored. Its use in different socio-cultural contexts, for example, is a challenge to overcome.

## Final remarks

The assessment of adolescent healthcare decisional capacity involves multiple intrinsic and extrinsic factors and requires professionals to recognize that decision-making extends beyond just the patient and the health professional. The theoretical approach to these factors underscores the need for a broad and multiprofessional evaluation, without which the autonomy of the adolescent patient risks being underestimated. While there is uncertainty about whether an assessment instrument can encompass all these factors, the academic community is challenged to design such a tool.

## Conflicts of interest

The authors declare no conflicts of interest.
